# A young woman with steroid‐responsive, IgG4‐positive plasma cell‐enriched cystic lymphangioma and chylous ascites

**DOI:** 10.1002/ccr3.1536

**Published:** 2018-04-17

**Authors:** Alaa A. Al Zaki, Shawna L. Mann, Mollie N. Carruthers, Graham W. Slack, Luke Y. C. Chen

**Affiliations:** ^1^ Division of Hematology Department of Medicine University of British Columbia Vancouver British Columbia Canada; ^2^ Division of Nephrology Department of Medicine University of Western Ontario London Ontario Canada; ^3^ Division of Rheumatology Department of Medicine University of British Columbia Vancouver British Columbia Canada; ^4^ Department of Pathology and Laboratory Medicine University of British Columbia Vancouver British Columbia Canada

**Keywords:** IgG4‐related disease, inflammation, lymphangioma, steroid

## Abstract

Lymphangiomas are benign tumors of the lymphatic vessels, which can be inflammatory and occasionally steroid‐responsive. IgG4‐related disease (IgG4‐RD) is a recently defined fibro‐inflammatory condition. We describe a novel association between reactive IgG4+ plasma cells and cystic lymphangioma in a young woman who had a dramatic clinical response to steroids.

## Case Report

A previously healthy 29‐year‐old Caucasian woman presented with a six‐month history of bilateral leg edema and weight loss (65–52 kg) associated with anorexia and nonbloody diarrhea. She denied any constitutional or infectious symptoms and had no history to suggest heart failure, liver cirrhosis, pulmonary or renal disease, such as dyspnea, frothy urine, abdominal pain, or jaundice. She was not on any prescription medications or recreational drugs. Her complete blood count, renal, and liver profiles were normal. C‐reactive protein was 57 mg/L (<3.1), albumin 7 g/L (34–50), and urinary protein 0.23 g/day. Antinuclear and antitissue transglutaminase antibodies, blood, urine, stool cultures, parasite examination, and viral serologies were all negative. Echocardiogram was normal. Computed tomography of the neck, chest, abdomen, and pelvis showed extensive retroperitoneal cystic lesions extending from the subdiaphragmatic region to the pelvic inlet, two discrete cysts in the left supraclavicular fossa, and mediastinum which appeared continuous via the thoracic duct suggestive of multiple lymphoceles. Upper and lower endoscopies and small bowel capsule studies were normal. Needle biopsies of the retroperitoneal and neck lesion were nondiagnostic.

**Figure 1:**
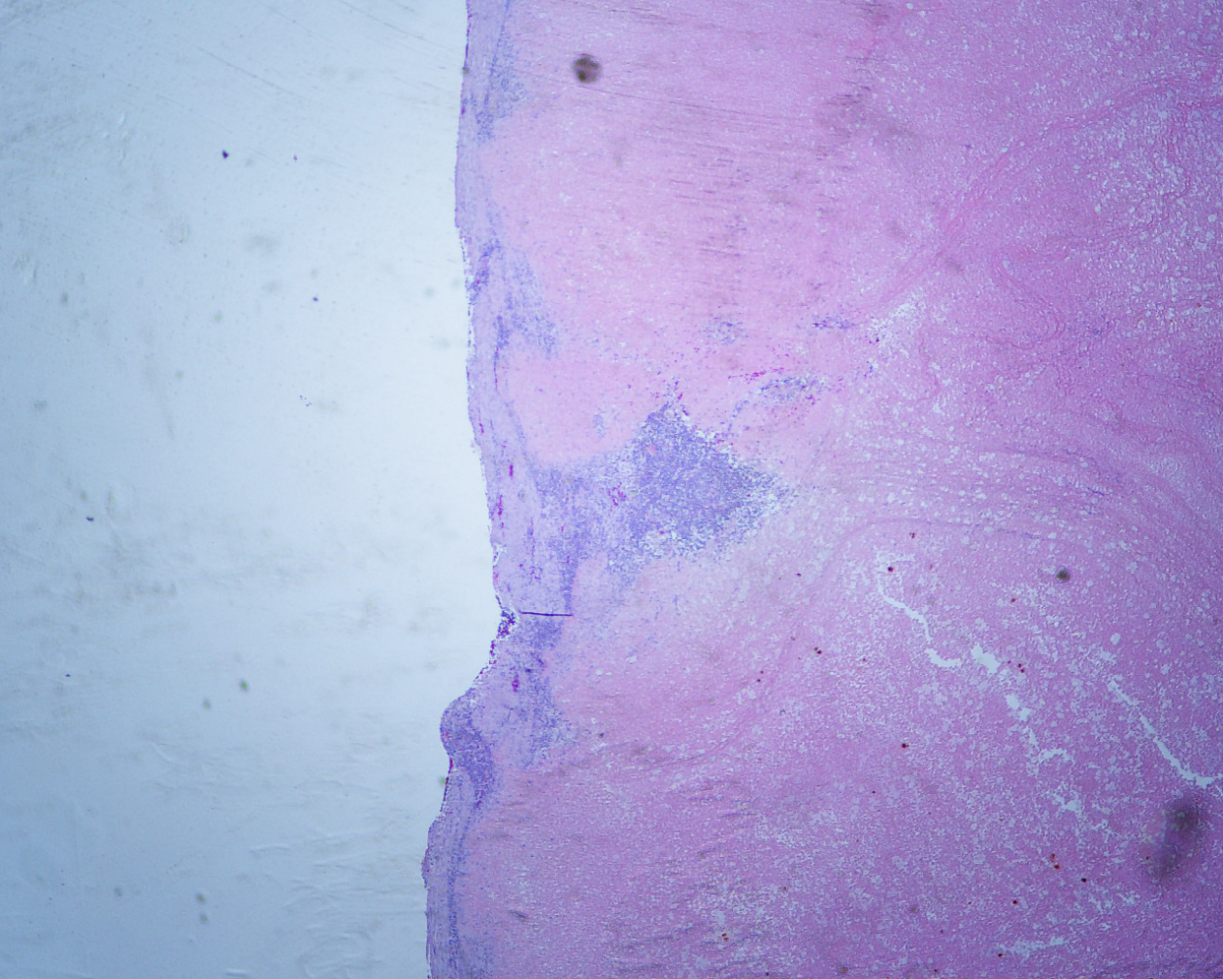
The pelvic mass is composed of necrotic tissue surrounded by a thin rim of fibrous tissue and chronic inflammation (hematoxylin–eosin, original magnification 20x).

**Figure 2:**
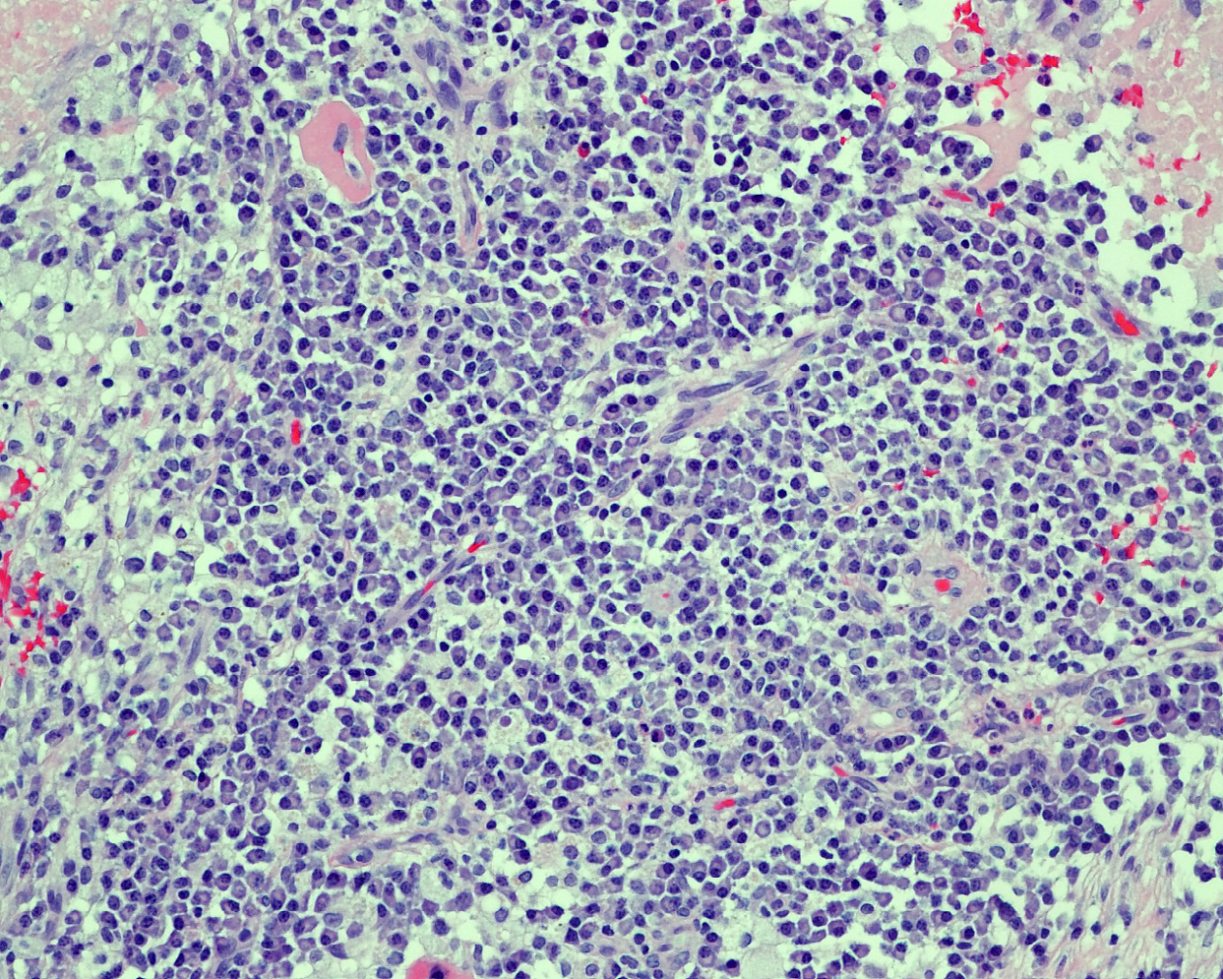
An immunohistochemical stain shows the vast majority (>95%) of plasma cells are positive for IgG4 (original magnification 200x).

**Figure 3:**
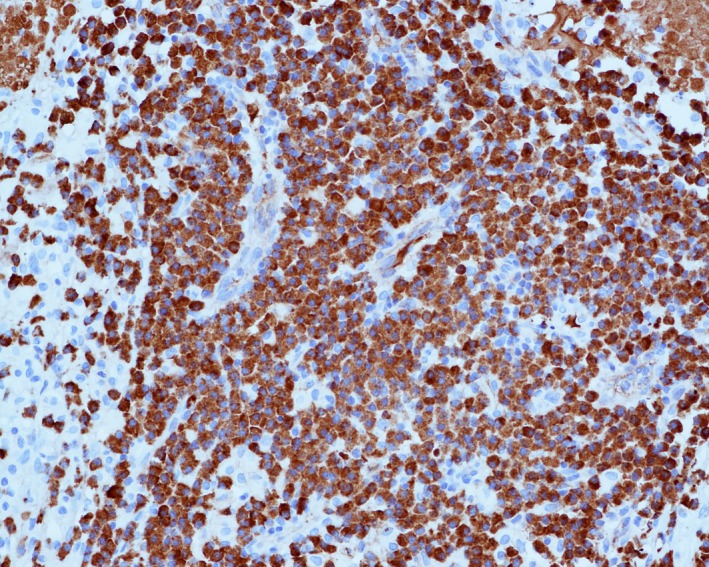
The chronic inflammatory infiltrate is made up of abundant small mature plasma cells (>100 per high power field) with a few admixed small mature lymphocytes and histiocytes (hematoxylin–eosin, original magnification 200x).

She developed anasarca and chylous ascites; after multiple inconclusive lymphangiographies, she underwent exploratory laparotomy with resection of the pelvic cysts. Pathology showed abundant plasma cells with >100IgG4+ cells/HPF and IgG4: IgG ratio 99% (Fig. 1 and 2). Other features of IgG4‐RD such as fibrosis, obliterative phlebitis, and eosinophilia were notably absent (Fig. 3). Bone marrow aspirate and biopsy showed some moderate hemophagocytosis, likely related to a recent blood transfusion, and otherwise normal trilineage hematopoiesis and a normal lymphoplasmacytic compartment. Serum protein electrophoresis, IgG (8.4 g/L), and IgG4 by immunonephelometry (0.44 g/L) were all normal.

Her ascites failed to improve with repeated drainage and total parenteral nutrition for 4 weeks. Although she did not meet diagnostic criteria for IgG4‐RD, the finding of IgG+ plasma cells led to a trial of systemic therapy with intravenous methylprednisolone 500 mg/day × 3 days 1. Her ascites improved dramatically by day 3, and she was switched to prednisone 50 mg daily. As prednisone was tapered to less than 10 mg daily, her ascites returned, so she was tried on numerous steroid‐sparing agents (rituximab, mycophenolate mofetil, azathioprine, and cyclosporine) with no appreciable effect. Finally, dietary modification (medium‐chain triglycerides and low‐fat diet) allowed control of her chylous ascites and gradual cessation of steroids.

## Discussion

Lymphangiomas are rare cystic malformation arising from sequestration of lymphatic tissue that fails to communicate with the lymphatic system. Ninety‐five percent cases are found in the neck and axilla. Retroperitoneal involvement constitutes only 1% 2. However, combinations of inflammatory, fibrotic, and genetic components have been postulated. Clinicopathological analysis of a series of intra‐abdominal cystic lymphangiomas demonstrated a tendency to induce marked inflammatory changes in the surrounding tissues characterized by florid myofibroblastic proliferation and occasionally xanthogranulomatous inflammation 3.

Immunoglobulin G4‐related disease (IgG4‐RD) is a systemic disease characterized by tumefactive lesions, particularly in glandular tissues (e.g., lacrimal and salivary glands, lymph nodes, and pancreas) and fibrosis. Nearly any organ or tissue can be involved, and the histology is remarkably similar in most tissues. Biopsies of affected organs demonstrate a lymphoplasmacytic infiltrate enriched with IgG4 + plasma cells (typically > 40% of IgG4/IgG ratio), storiform fibrosis, and obliterative phlebitis. Notable exceptions are bone marrow and lymph nodes, where fibrosis and obliterative phlebitis are not seen 4, and kidney, where two distinct patterns can be seen which are as follows: tubulointerstitial nephritis in 80% of cases and membranous glomerulonephritis in 20% 5. Approximately 70% of cases of patient have an elevated serum IgG4 level 6, and while most centers use immunonephelometry to measure serum IgG subclasses, mass spectrometry is a more accurate method 7. Despite the name, neither the presence of IgG4‐positive plasma cells in tissue nor the elevated serum IgG4 is specific for IgG4‐RD in the absence of other histological findings; diagnosis should be based on the International Consensus Criteria 1. Increased IgG4+ plasma cells may be seen in a number of “mimickers” of IgG4‐RD including Rosai‐Dorfman disease, multicentric Castleman's disease, and vasculitis. Increased serum IgG4, particularly mild increases in the 1.5–5 g/L range, may be seen in infection, autoimmune disease, hematologic neoplasms such as hypereosinophilic syndromes, and other conditions 8, 9. In this case, extensive review of the clinical, radiologic, and pathologic features did not confirm a diagnosis of IgG4‐RD, and the IgG4 plasma cell infiltrate appears to be reactive in nature. However, the finding of IgG4+ plasma cells led to the hypothesis that her symptoms might respond to steroids, which are the first‐line therapy in IgG4‐RD. Her dramatic response to steroids confirms a significant inflammatory component to her underlying disease process. This case invites further exploration of a potential pathophysiological link between IgG4‐RD and cystic lymphangioma.

## Authorship

AAAZ: was the first author, wrote the main body of the manuscript, and performed thorough literature search to enrich the discussion part. SLM: was the second author, gathered information, medical history, and physical examination, and contributed to the literature search and manuscript editing. MNC: was the third author, contributed with rheumatology investigation of the case and management of IgG4‐related inflammatory infiltrate with steroid, and contributed to manuscript editing and IgG4 RD discussion. GWS: was the fourth author, provided high‐quality microscopic images of the biopsy with the histopathological description, and contributed to manuscript editing. LYCC: was the fifth author and main treating physician of the patient, supervised manuscript writing and editing, and contributed in writing the discussion part. All authors had access to the data and participated in the writing of the manuscript.

## Conflict of Interest

None of the authors have any conflict of interest or financial disclosures related to this manuscript.
